# Bottom-Water Conditions in a Marine Basin after the Cretaceous–Paleogene Impact Event: Timing the Recovery of Oxygen Levels and Productivity

**DOI:** 10.1371/journal.pone.0082242

**Published:** 2013-12-13

**Authors:** Claudia Sosa-Montes De Oca, Francisca Martínez-Ruiz, Francisco Javier Rodríguez-Tovar

**Affiliations:** 1 Instituto Andaluz de Ciencias de la Tierra, Consejo Superior de Investigaciones Científicas-Universidad de Granada, Armilla, Granada, Spain; 2 Departamento de Estratigrafía y Paleontología, Universidad de Granada, Granada, Spain; University of Florence, Italy

## Abstract

An ultra-high-resolution analysis of major and trace element contents from the Cretaceous–Paleogene boundary interval in the Caravaca section, southeast Spain, reveals a quick recovery of depositional conditions after the impact event. Enrichment/depletion profiles of redox sensitive elements indicate significant geochemical anomalies just within the boundary ejecta layer, supporting an instantaneous recovery –some 10^2^ years– of pre-impact conditions in terms of oxygenation. Geochemical redox proxies point to oxygen levels comparable to those at the end of the Cretaceous shortly after impact, which is further evidenced by the contemporary macrobenthic colonization of opportunistic tracemakers. Recovery of the oxygen conditions was therefore several orders shorter than traditional proposals (10^4^–10^5^ years), suggesting a probable rapid recovery of deep-sea ecosystems at bottom and in intermediate waters.

## Introduction

The Cretaceous–Paleogene boundary (K/Pg), ≈65.95 Ma ago, is marked by one of the major faunal extinctions during the Phanerozoic, which led to the disappearance of about 70% of existing marine and continental species [1]. In particular, more than 90% of Maastrichtian planktic species of foraminifera disappeared abruptly at this boundary [2], [3]. The hypothesis of an extraterrestrial impact [4], [5] to explain the extinction has been widely accepted [6], though some authors also relate this mass extinction event with the activity of the large igneous province of the Deccan Traps [7]–[9], and debate goes on in the literature regarding volcanism, impacts and mass extinctions [10]. However, the synchroneity of a bolide impact and the associated mass extinction has been demonstrated [11]. Despite intensive research, many open questions remain; how and when biological productivity recovered after the impact, and how different ecosystems responded to such environmental changes are still controversial matters. Further understanding of the response of marine ecosystems to global catastrophes calls for deeper study of environmental conditions across this boundary.

The Chicxulub impact [12], [13] involved a large bolide, about 10±4 km in diameter, that produced severe effects at the local and regional scale [14], including earthquakes of magnitude >11 causing continental and marine landslides, tsunamis of 100–300 m in height that swept more than 300 km onshore and carried continental debris basin-ward to deep-marine sequences, shock waves and air blasts that radiated across the landscape, and high temperatures that generated fires within distances of 1,500 to 4,000 km from the crater. It is estimated that instantaneous combustions of 18%–24% of the terrestrial biomass existed at that time [15]. Other global effects were nitric and sulfuric acid rain, widespread dust and blackout that prevented sunlight from reaching the surface of the Earth and lowered its temperature, and destruction of the stratospheric ozone layer, with a greenhouse effect that led to a temperature increase of 1.5° to 7.5°C. Hence, the impact generated an initial brief warming, followed by a short cooling period (≈2 kyr) and then a warm phase [14].

In addition to the major extinction, these environmental changes led to severe changes in depositional conditions, particularly in marine basins. Constraining how fast such conditions recovered is essential to further understand the recovery of ocean productivity, and how ecosystems adapt to major environmental changes. As a result of organic matter and metal accumulations, bottom waters underwent severe oxygen depletion. Trace metal anomalies point to major changes in redox conditions across the boundary [16], also indicated by biomarkers [17]. In this regard, the basal 3-mm-thick K/Pg layer at Caravaca section (Southeast Spain) shows a rapid increase in terrestrial long-chain *n*-alkanes and dibenzofuran, signaling a greater supply of terrestrial organic matter as well as a rapid increase in the concentration of dibenzothiophenes, evidencing a change from oxic to anoxic/euxinic conditions in the intermediate water above the seafloor [17]. Similarly, inorganic redox proxies [18], [19] allow us to reconstruct the evolution of oxygen conditions.

This study focuses on the Caravaca section, one of the best-preserved sections worldwide, well exposed and continuous [20]. It has been extensively studied during the last three decades [5], [17], [21]–[23], and can be considered a highly representative distal section for analysis of the K/Pg impact event. Selection of the sampling interval based on absence of mixing and traces fossils across the boundary to ensure that sampling at a millimetric scale records the original distribution of geochemical signatures. Hence, we present a mm-scale resolution approach, based on geochemical proxies in combination with icnological data, to gain insight into the timing of oxygen recovery and the recovery of biological productivity after the impact event.

## Geologic Setting

**Figure 1:**
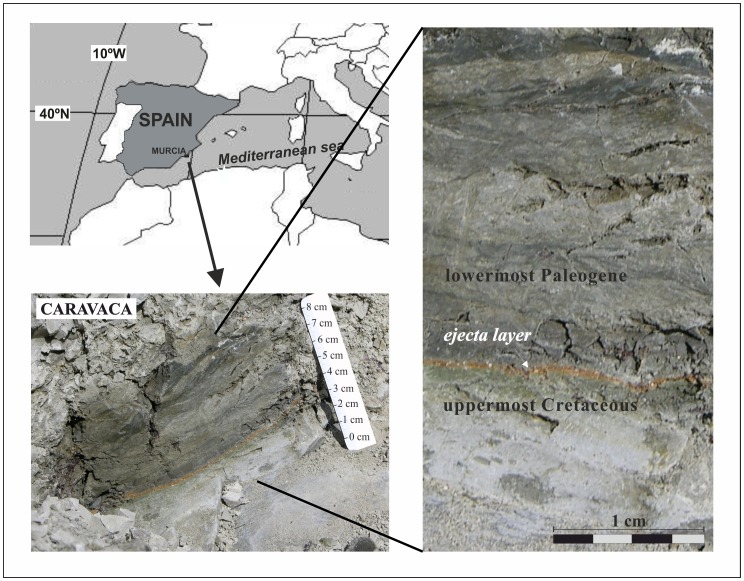
Caravaca outcrop. Location and close-up photographs of the Cretaceous–Paleogene (K/Pg) boundary section at Caravaca (Southeast Spain).

The K/Pg boundary section at Caravaca (38°04′36.39″N, 1°52′41.45″W) is located on the NW side of road C-336, about 4 km southwest of the town of Caravaca (Murcia, Spain), in the Barranco del Gredero (Figure 1). The studied section belongs to the external Subbetic of the Betic Cordillera. Lithology consists of light marls in the upper levels of the Maastrichtian sediments (uppermost Cretaceous), followed by 7–10 cm of a lower Danian (lowermost Paleogene) blackish gray clay layer (the so-called boundary clay layer) with a 2–3 mm thick reddish brown layer at the base (ejecta layer) containing spherules and platinum group element (PGE) anomalies [16], [24]–[26]. The 7–10 cm lower Danian clay layer gradually increases its carbonate content to a gray argillaceous marl similar to that of the upper Cretaceous (Figure 1). The Caravaca section, like the nearby Agost section (115 km away, in Alicante, Spain) and the El Kef section (Tunisia), is one of the best-preserved distal sections in the world [27]. It is thought to represent deposition at paleowater depths of ∼200 to 1,000 m [28], [29] and at around 27–30°N paleolatitude [29]–[31].

## Materials and Methods

In the framework of mm-scale resolution analysis across the K/Pg boundary, we focused on a 4.20 cm interval, from 1.20 cm below the K/Pg boundary to 3.0 cm above it, recording depositional conditions at the Latest Cretaceous, those of the ejecta layer, and the Earliest Danian. The fieldwork was carried out in public land and no specific permission was required. Samples were taken in continuous sampling every 0.2 cm. Ichnological analysis revealed a well-developed lowermost Danian trace fossil assemblage, even penetrating vertically into the Cretaceous sediments. Nonetheless, a careful selection of sampled intervals was done to avoid disturbation across the boundary. Thus, this highly detailed sampling involved materials showing no evidence of discrete trace fossils and without any mixing by bioturbation. According to the sedimentation rates of 3.1 cm Kyr^−1^ estimated for the Maastrichtian sediments, and that of 0.8 cm Kyr^−1^ calculated for the boundary clay layer [23], the studied material would span a time interval from 400 years prior to the K/Pg boundary to 3,750 years afterward.

Major and trace element concentrations were respectively obtained by Atomic Absorption Spectrometry (AAS) and Inductively Coupled Plasma Mass Spectrometry (ICP-mass), at the Centre for Scientific Instrumentation (CIC), University of Granada, Spain. All samples were crushed in an agate mortar and digested with HNO_3_+HF [32].

We used Al-normalized concentrations of redox sensitive elements (V/Al, Mo/Al, U/Al, Pb/Al, Ni/Al, Co/Al, Cu/Al, Zn/Al and Cr/Al ratios), the U/Mo ratio, authigenic factors (Aut), and enrichment factors (EFs) of U and Mo for the reconstruction of redox conditions.

Enrichment factors (EF) were calculated as:
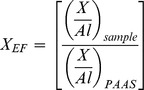



Authigenic factors (aut) were calculated as:
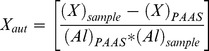



where X and Al represent the weight percentage concentrations of elements X and Al, respectively. Samples were normalized using post-Archean average shale (PAAS) compositions [33].

Rare earth element (REE) concentrations were also determined in order to show the nature of the ejecta layer regarding sediments deposited above and below the K/Pg boundary [16].

## Results and Discussion

It is well known that the K/Pg boundary marks major changes in the chemical composition of sediments deposited across it. Some changes can be expected as a consequence of the sudden drop in carbonate production, and the subsequent change in sediment lithology. Geochemical changes across the boundary are particularly evident in distal sections where a significant extraterrestrial metal contribution is recognized. In contrast, at sections located closer to the impact site, such as Blake Nose [34] or Demerara Rise [35] in the Western Atlantic, the extraterrestrial metal contribution is highly diluted by target rocks.

In distal sections, as the one here studied, the bolide contribution together with the enhanced chemical alteration in emerged areas produced a high metal supply. Additionally, reduced oxygen levels, due to the greater input of organic matter (both terrestrial and marine), also promoted anomalous concentrations of trace elements across the K/Pg boundary [13], [16], [21]. Despite diagenesis and potential remobilization, original signatures are preserved, evidenced by PGE anomalies [16], [24]–[26] and the extraterrestrial nature of trace elements such as Cr [36] within the ejecta layer. After the ejecta deposition, the autochthonous terrigenous supply led to the deposition of the boundary clay; primarily as a consequence of the reduced carbonate production. Therefore, the ejecta layer and this clay layer record the impact and post-impact depositional conditions, respectively. Impact evidence at Caravaca section also includes diagenetically altered spherules, largely composed of smectites and K-feldspar [37].

**Figure 2:**
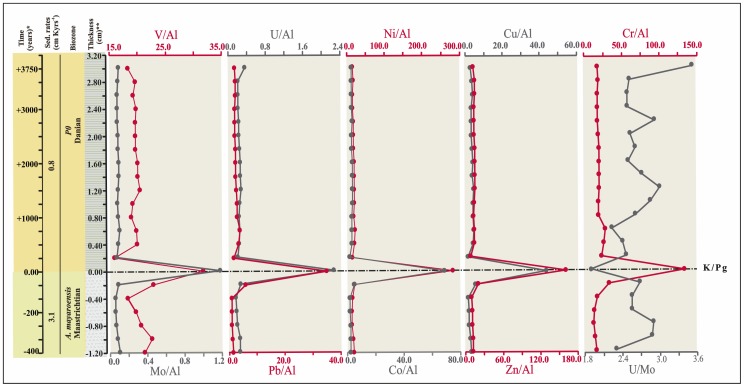
Paleoredox proxy ratios. Enrichment/depletion profiles of redox-sensitive elements across the Cretaceous–Paleogene (K/Pg) boundary at Caravaca section (Al normalized concentrations *10^−4^). *The time (years) was calculated base on sedimentation rates in Kaiho et al. [23], before and after K/Pg event. **Thickness (cm) from K/Pg event.

**Figure 3:**
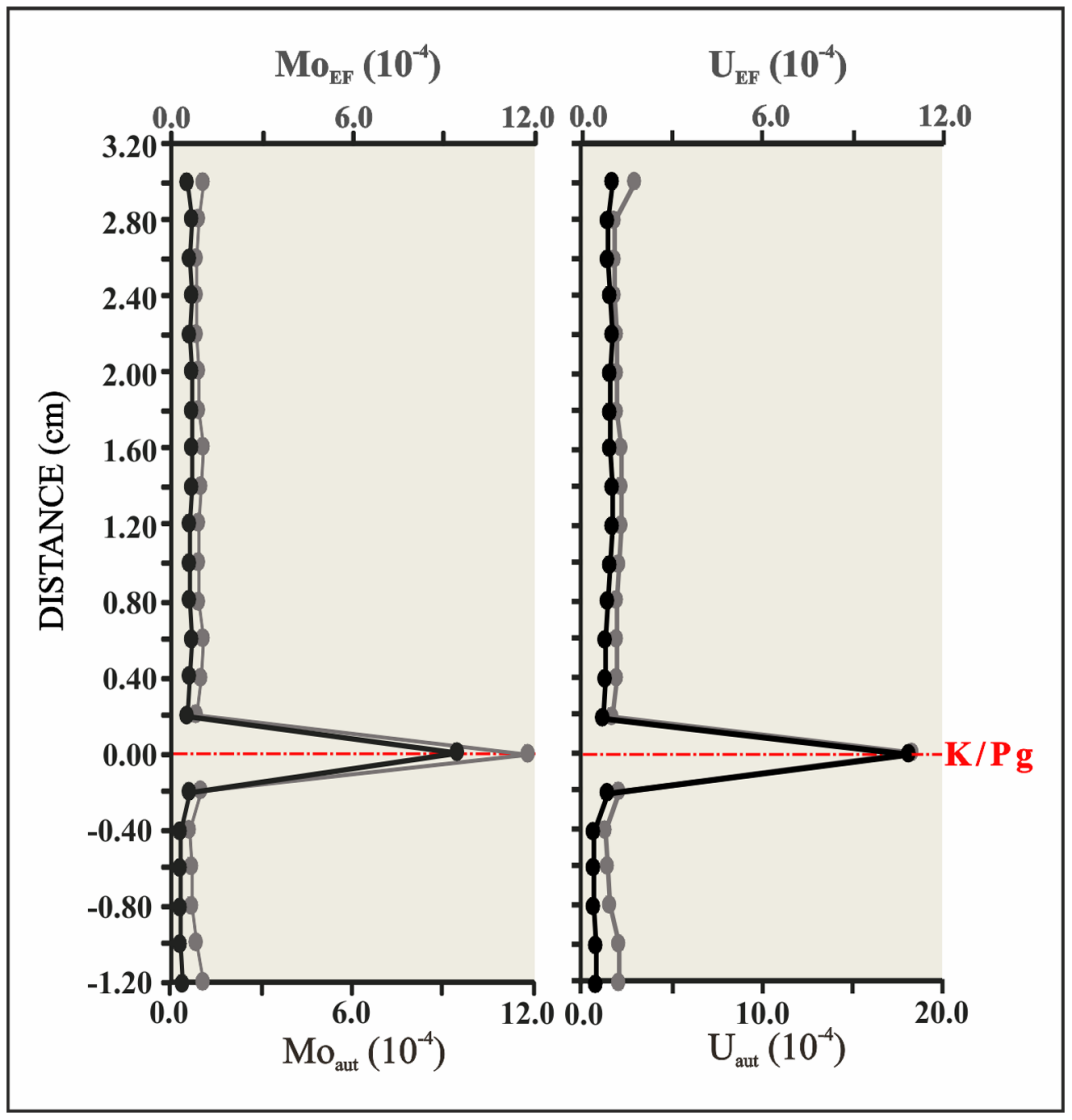
Mo_EF-aut_ and U_EF-aut_ variations. Profiles Mo_EF-aut_ and U_EF-aut_ for the Cretaceous–Paleogene (K/Pg) boundary section at Caravaca (Southeast Spain).

**Figure 4:**
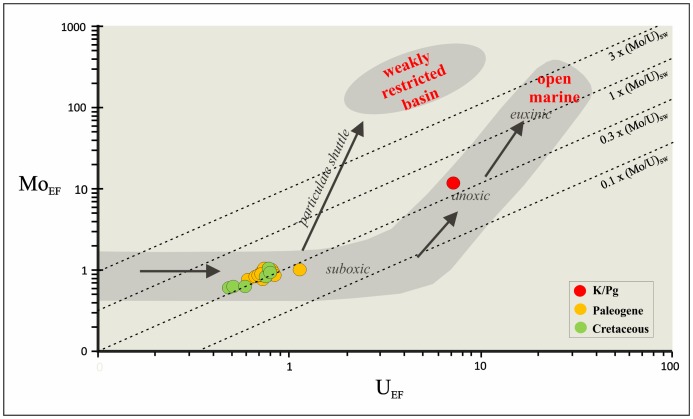
Mo_EF_ vs U_EF_ covariation. Mo_EF_ vs U_EF_ covariation for the Cretaceous–Paleogene (K/Pg) boundary section at Caravaca (Southeast Spain).

**Figure 5:**
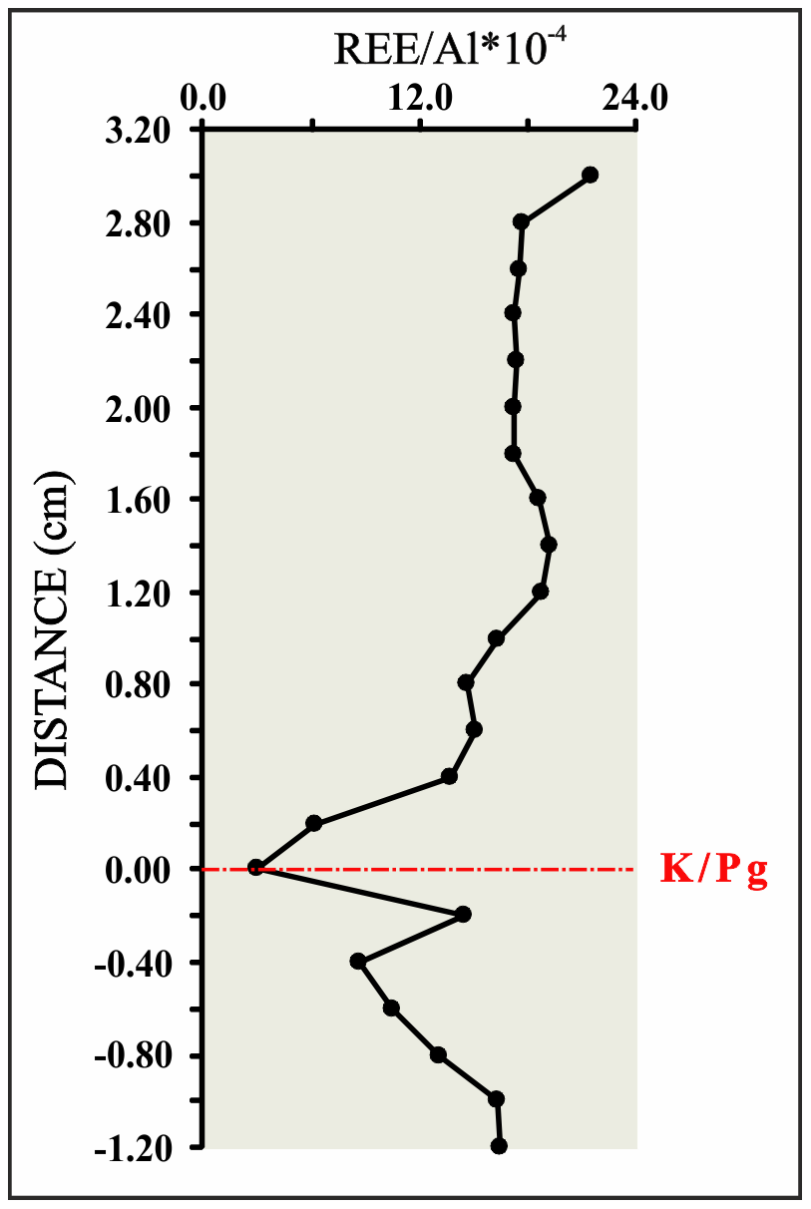
REE variation profile. REE/Al ratio (Al normalized concentrations *10^−4^) for the Cretaceous–Paleogene (K/Pg) boundary section at Caravaca (Southeast Spain).

Our mm-scale resolution analysis of trace metal concentrations and elemental ratios (V/Al, Cr/Al, Co/Al, Ni/Al, U/Al, Cu/Al, Zn/Al, Mo/Al, Pb/Al, and U/Mo) support the significant geochemical anomalies of the ejecta layer (Figure 2). These ratios sharply peak just within the ejecta layer, with values (*10^−4^) of 31.65 for the V/Al ratio, 1.18 for the Mo/Al ratio, 2.25 for the U/Al ratio, 280.50 for the Ni/Al ratio, 34.98 for the Pb/Al ratio, 69.02 for the Co/Al ratio, 42.95 for the Cu/Al ratio, 160.70 for the Zn/Al ratio, and 132.16 for the Cr/Al ratio; in contrast, the U/Mo ratio (1.91) (Table 1) [38] shows a noteworthy depletion. The abundance of U and Mo is a particularly useful proxy for paleoredox conditions [39], [40]. Significant enrichments of U and Mo in marine sediments may generally be imputed to authigenic uptake of these elements from seawater in suboxic (for U) or euxinic conditions (for Mo) (Figure 3). The decrease in the U/Mo ratio thus suggests that sulfidic conditions at this time may have favored a major Mo uptake. The U_FE_ vs Mo_FE_ covariation (Figure 4) also indicates a change in redox conditions just within the K/Pg boundary, which implies a quick return to previous Cretaceous oxygen levels after the impact. Yet a comparison of redox proxies (Figure 2) between Late Cretaceous sediments and those deposited during the very Early Danian showed no major changes, which suggests that oxygenation conditions during the Early Danian were not dramatically different from pre-impact conditions. On a global scale, no evidence of global hypoxia is reported, only rather low oxygen conditions at a local scale for certain outcrops [41]. Our data therefore support that lower oxygenation was mostly restricted to the deposition of the ejecta layer, that was settled down instantly on a geological time scale [42], while sediments from the Early Danian and the Late Cretaceous are similar in terms of oxygenation. The distinct nature of the ejecta layer is moreover supported by the REE depletion (Figure 5), derived not only from the diagenetic alteration of the impact glass and subsequent loss of REE, but also from the relatively high contribution of REE-depleted extraterrestrial material [16], [21].

**Table 1:** Element content and elemental ratios.

SAMPLE	DISTANCE(cm)	Al	Ca	V/Al	Mo/Al	U/Al	Pb/Al	Ni/Al	Co/Al	Cu/Al	Zn/Al	Cr/Al	REE/Al	U/Mo	Mo_EF_	U_EF_	Mo_aut_	U_aut_
CA +2.8+3.0	3.00	5.25	18.94	18.31	0.10	0.36	2.34	15.29	3.45	3.20	14.37	17.39	21.57	3.53	1.01	1.15	0.48	1.71
CA +2.6+2.8	2.80	8.17	6.16	19.56	0.09	0.22	2.62	17.47	3.71	4.17	16.03	18.81	17.78	2.52	0.87	0.71	0.63	1.54
CA +2.4+2.6	2.60	8.40	6.13	19.21	0.08	0.21	2.50	16.13	3.50	4.55	16.44	17.58	17.49	2.49	0.84	0.68	0.63	1.51
CA +2.2+2.4	2.40	8.69	5.67	19.72	0.09	0.21	2.19	16.32	3.57	3.73	15.31	17.48	17.28	2.49	0.85	0.68	0.65	1.57
CA +2.0+2.2	2.20	8.50	6.59	19.57	0.08	0.23	2.36	16.92	3.74	3.67	15.31	17.99	17.36	2.93	0.79	0.74	0.59	1.70
CA +1.8+2.0	2.00	8.37	6.99	19.58	0.09	0.23	2.36	16.87	3.65	3.84	15.44	18.51	17.29	2.53	0.90	0.73	0.67	1.64
CA +1.6+1.8	1.80	8.22	7.28	19.59	0.09	0.24	2.54	18.09	3.59	4.25	15.74	19.06	17.28	2.62	0.90	0.76	0.66	1.69
CA +1.4+1.6	1.60	7.40	8.31	19.92	0.10	0.25	2.65	19.03	3.91	4.43	16.26	19.96	18.58	2.51	1.01	0.82	0.68	1.65
CA +1.2+1.4	1.40	7.50	7.74	19.90	0.10	0.26	2.62	18.29	3.81	4.30	15.83	19.92	19.30	2.72	0.96	0.84	0.65	1.73
CA +1.0+1.2	1.20	7.56	8.51	20.36	0.09	0.27	2.88	19.22	3.86	4.75	15.90	19.99	18.76	3.00	0.89	0.86	0.59	1.78
CA +0.8+1.0	1.00	7.56	8.01	19.03	0.09	0.25	3.02	18.20	4.07	4.55	14.94	18.88	16.36	2.86	0.86	0.79	0.57	1.63
CA +0.6+0.8	0.80	7.69	7.31	18.78	0.09	0.23	3.13	16.49	3.56	4.53	14.34	18.26	14.70	2.61	0.87	0.73	0.59	1.51
CA +0.4+0.6	0.60	6.95	7.38	19.69	0.11	0.24	3.89	20.40	3.61	4.88	14.79	27.77	15.17	2.25	1.05	0.76	0.66	1.42
CA +0.2+0.4	0.40	6.95	8.84	19.84	0.09	0.23	3.63	18.33	3.45	4.68	15.57	25.24	13.76	2.42	0.93	0.73	0.58	1.35
CA +0.0+0.2	0.20	7.44	6.78	15.70	0.08	0.19	1.75	13.35	1.95	1.73	9.07	22.78	6.25	2.47	0.78	0.62	0.51	1.20
**CA K/Pg**	**0.00**	**8.11**	**6.57**	**31.65**	**1.18**	**2.25**	**34.98**	**280.50**	**69.02**	**42.95**	**160.70**	**132.16**	**3.02**	**1.91**	**11.80**	**7.25**	**9.49**	**17.99**
CA 0.0 −0.2	−0.20	6.70	12.52	22.76	0.09	0.25	6.08	19.12	4.78	5.71	21.46	31.97	14.43	2.68	0.94	0.81	0.56	1.48
CA −0.2 **−**0.4	**−**0.40	5.95	13.38	18.13	0.06	0.15	1.17	12.95	2.10	1.91	10.41	16.53	8.63	2.56	0.60	0.50	0.30	0.74
CA **−**0.4 **−**0.6	−0.60	5.38	23.48	19.60	0.06	0.16	1.07	12.32	2.08	2.15	11.53	12.59	10.58	2.56	0.63	0.52	0.29	0.70
CA −0.6 −0.8	−0.80	4.67	21.78	20.49	0.06	0.19	1.06	12.41	2.18	2.35	11.91	11.14	13.15	2.90	0.64	0.60	0.25	0.73
CA −0.8 −1.0	−1.00	4.12	23.95	22.42	0.08	0.24	1.31	15.76	2.51	2.81	12.10	13.82	16.28	2.88	0.83	0.77	0.30	0.85
CA −1.0 −1.2	−1.20	3.89	24.40	21.07	0.11	0.25	1.56	18.06	2.55	3.36	12.66	15.59	16.49	2.31	1.08	0.80	0.38	0.85

Al and Ca concentrations (%), and elemental ratios (*10^−4^) across the Cretaceous–Paleogene (K/Pg) boundary at the Caravaca section.

In view of the distribution profiles of trace metals in terms of timing, and the interval where pre-impact concentrations were reached (occurring at a distance between 0.2 and 0.3 cm above the K/Pg boundary), as well the sedimentation rates of the first centimeters of the Danian clay –0.8 cm kyr^−1^ in Caravaca [23]– we infer that oxygenation conditions were recovered in less than 375 years (in the order of 10^2^ years). This value is several orders less than intervals traditionally proposed (10^4^–10^5^ years) [22], [23]. Such timing differences with respect to previous works may derive from a much higher resolution sampling. Furthermore, the reconstruction of oxygen conditions was based on recently developed geochemical redox proxies that have proven to be reliable [36], [37], [38]. Accordingly, our data support that oxygenation conditions recovered very quickly, almost instantly on a geological time scale [42].

Such a conclusion is also in agreement with the rapid recovery interpreted for the macrobenthic tracemaker community based on the presence of Fe-oxide spherules in the infilling of *Thalassinoides* in the Agost section [43], and with the bioturbational disturbance of the 2–3-mm-thick K/Pg red boundary layer at the Caravaca section [44]. In the latter case, the bioturbational disturbance is produced by *Chondrites* and *Zoophycos* tracemakers, favored by their relative independence from substrate features, together with an opportunistic behavior allowing colonization of sediment poor in oxygenated pore waters and food [44]–[46]. Therefore, the geochemical results reported here and previous ichnological data both support that the recovery to normal levels of seafloor oxygenation was almost instantaneous, with absolute values lower than 10^2^ years.

The recovery of planktic foraminifera has been linked to that of the marine carbon system. The evolutionary recovery and biogeochemical recovery occurred in two stages, up to four million years after the extinction [47]. This is conditioned by the extremely long time (millions of years) required to repair food chains and to reestablish an integrated ecosystem [48].

In the benthic environment, benthic foraminifera reflect no major extinction at the K/Pg boundary, regardless of whether they were shallow or deep dwellers, high or low latitude forms, or infaunal or epifaunal inhabitants [49]. However, diversity of the assemblages and number of infaunal morphogroups decreased severely [41]. The recovery of these benthic foraminifera assemblages took a few thousand to a few hundred thousand years, suggesting the inhabitability of the benthic foraminifera habitat, and that their food supply likewise did not fully recover during the first few hundred thousand years after impact [49]. In the macrobenthic habitat, however, we surmise that macrobenthic opportunist taxa could have initiated a very quick recovery, since low oxygen and adverse environmental conditions were reinstated shortly after the impact. Although full faunal recovery to pre-extinction abundances and the complete recovery of the marine carbon system occurred over a much longer period [48], the earliest response of oceanic ecosystems to the largest biotic disturbance of the Cenozoic in terms of timing was most likely very rapid.

## Conclusions

A mm-scale resolution geochemical analysis across the K/Pg boundary at the Caravaca section evidences a rapid return to pre-impact conditions in terms of oxygenation after this major catastrophe. According to the estimated sedimentation rates for this section, oxygen levels at bottom and intermediate waters recovered at a very fast rate, in a range of few hundred years after the K/Pg boundary event. Depositional conditions for the ejecta layer were highly anoxic, as a consequence of the enhanced contribution of metals to the basins, accompanied by a greater supply of terrestrial and marine organic material. However, shortly after the impact, oxygen levels rapidly recovered, favoring the earliest macrobenthic opportunist colonization.
